# Small-diameter bacterial cellulose-based vascular grafts for coronary artery bypass grafting in a pig model

**DOI:** 10.3389/fcvm.2022.881557

**Published:** 2022-09-26

**Authors:** Deborah Fusco, Florian Meissner, Bruno K. Podesser, Anna Marsano, Martin Grapow, Friedrich Eckstein, Bernhard Winkler

**Affiliations:** ^1^Department of Biomedicine, University of Basel, Basel, Switzerland; ^2^Department of Cardiac Surgery, University Hospital Basel, Basel, Switzerland; ^3^Center for Biomedical Research, Medical University Vienna, Vienna, Austria; ^4^Heart Center Hirslanden Zurich, Zurich, Switzerland; ^5^Department of Cardiovascular Surgery, Vienna Heart Center KFL, Vienna, Austria

**Keywords:** coronary artery, vascular graft, cellulose, surgery, tissue engineering

## Abstract

Surgical revascularization is the gold standard in most cases of complex coronary artery disease. For coronary artery bypass grafting, autologous grafts are state-of-the-art due to their long-term patency. A non-negligible amount of patients lack suitable bypass material as a result of concomitant diseases or previous interventions. As a promising alternative, tissue-engineered vascular grafts made of biomaterials such as bacterial cellulose (BC) are gaining more and more attention. However, the production of small-diameter grafts (inner diameter < 6 mm) of application-oriented length (> 5 cm) and their *in vivo* long-term patency remain challenging. In this study, grafts of 20 cm in length with an inner diameter of 3 mm were generated in a custom-made bioreactor. To potentially improve graft compliance and, therefore *in vivo* patency, BC was combined with an embedded cobalt–chromium mesh. The grafts were designed for *in vivo* endothelialization and specific surgical properties and implanted as an aortocoronary bypass in a left anterior descending occluded pig model (*n* = 8). Coronary angiography showed complete patency postoperatively at 4 weeks. Following 4 weeks *in vivo*, the grafts were explanted revealing a three-layered wall structure. Grafts were colonized by smooth muscle cells and a luminal layer of endothelial cells with early formation of *vasa privata* indicating functional remodeling. These encouraging findings in a large animal model reveal the great potential of small-diameter BC grafts for coronary and peripheral bypass grafting.

## Introduction

Coronary and peripheral vascular diseases are the leading cause of death worldwide (1). Every year more than 350,000 people die in the USA because of coronary artery disease (2). In the case of complex multivessel coronary artery disease, myocardial revascularization by coronary artery bypass grafting (CABG) is the state-of-the-art therapeutic procedure. Commonly, CABG requires autologous bypass grafts such as the internal thoracic artery, the great saphenous vein, and the radial artery. Their availability is restricted due to multivascular diseases, poor graft quality, and previous surgeries. Furthermore, the long-term patency of vein grafts seems to be limited and inferior when compared with arterial grafts (3). Moreover, a second surgical site increases the risk of complications such as wound infection, postoperative pain, and bleeding. For large-diameter peripheral bypass grafting, vascular prostheses made of Dacron^®^ or polytetrafluoroethylene (PTFE) are commercially available. When applied as small-diameter vascular grafts (inner diameter < 5 mm), these materials showed an increased risk of thrombosis and intimal hyperplasia, resulting in a poor *in vivo* long-term patency (4). These findings restricted the use of these materials as well as the use of cryopreserved homografts as early as 1980. Various studies were undertaken with the result that these materials were left for the small diameter application and scientific interest was drawn toward engineering different forms of artificial grafts (5, 6). The patency rates varied but in conclusion showed a dramatic low rate at 12 months of up to 80% failure rate depending on the implantation site. For peripheral applications, the rates remained slightly higher at 40% patency after 1 year (5). Since the early days of cardiac surgery, tissue-engineered biomaterials grafts (TEVG) made of various biomaterials have been considered a promising alternative. As one prominent example, antithrombotic vascular grafts made of extracted human fibroblasts were implanted successfully *in vivo* from Cytograft Tissue Engineering Inc. (Novato, USA) (7). However, in clinical practice, no TEVG has been implemented for arterial bypass grafting. Another favorable biomaterial used for vascular grafts is bacterial cellulose (BC), which is excreted extracellularly by certain bacteria species, namely AcetoBacter Xylinum (ABX) (8). This biomaterial has been used for many biomedical applications like skin substitutes and is now explored for TEVGs (9). In addition to its biocompatibility and unique biomechanical properties, its nanofibrillar structure allows vascular remodeling by endothelial cells (ECs) and smooth muscle cells (SMCs). Preliminary results from studies applying BC grafts for peripheral bypass grafting in large animal models are promising, although they are limited by the restricted *in vivo* long-term patency (10, 11).

Since the limited *in vivo* patency of previous BC grafts might be related to a compliance mismatch between the graft and the native vessel, potential modifications of BC graft compliance have been discussed (12).

In the past, manufacturing small-diameter BC-vascular grafts (inner diameter < 6 mm) of application-oriented length (> 5 cm) has been challenging. Furthermore, these grafts have not been applied as coronary artery bypass grafts in a large animal ischemic heart model so far. Therefore, this study aimed to generate a small-diameter BC-vascular graft of more than 15 cm in length for CABG in a pig model as a proof of concept study.

## Materials and methods

### Generation of bacterial cellulose-vascular grafts

#### Cultivation of AcetoBacter xylinum

The bacterial strain used to produce BC was ABX subsp. sucrofermentas Toyosaki et al. (ATCC^©^ 700178*™*). Deep-frozen cultures were stored at –70°C. Hestrin–Schramm medium containing glucose 50% (2.0% w/v), yeast extract (0.5%), proteose peptone (0.5%), Na_2_HPO_4_ (0.27%), and citric acid (0.115%) was used for cultivation. The cultivation medium containing suspended ABX was incubated at 26°C for 18 days under static conditions at room oxygen.

#### Bioreactor-based culture of the reinforced bacterial cellulose-vascular grafts

Bacterial cellulose (BC)-vascular grafts are generated in a customized open rotating bioreactor (Supplementary Figure 1). The bioreactor contains four independent units. Each unit consists of a horizontally rotating steel shaft (outer diameter = 2 mm) covered by a silicone tube (outer/inner diameter = 3/2 mm). For BC fermentation, glucose-based medium containing glucose 50% (2.381% w/v), KH_2_PO4 (0.7%), MgSO_4_ × 7 H_2_O (0.213%), H_3_BO_3_ (0.00043%), nicotinamide (0.00007%), FeSO_4_ × 7 H_2_O (0.00095%), Na_2_HPO_4_ (0.134%), (NH_4_)_2_SO_4_ (0.354%), and ethanol (0.473%) was used. The bioreactor was filled with bacterial suspension (fermentation media + bacteria) up to the level of the silicone-covered steel shafts. Each unit was controlled by a stepper motor with a rotational speed of 30 rpm. BC fermentation process lasts 5 days at 26–28°C. BC is produced at the air-liquid interface by Acetobacter Xylinus. Steel shaft rotation is necessary for wrapping the BC layers around the silicon tube over the fermentation time. After 2.5 days of culture, the steel shafts with the preliminary BC grafts were removed from the bioreactor. Knitted VEST^®^ cobalt–chromium meshes from Vascular Graft Solutions (VGS, Tel-Aviv, Israel, outer-diameter 5 mm) were placed over the grafts and incubated at 26–28°C for 2.5 days more. After harvesting, the grafts were purified from bacteria in NaOH solution for 11 days. On day 8, each graft was heated up to 60°C for 4 h. For storage, the grafts were placed horizontally in sterile glass tubes at 2–8°C in standard sodium chloride solution after being autoclaved at 121°C in ddH_2_O.

### *In vitro* assessments

#### Micro-computed tomography

Micro-CT was applied to determine the mesh position along the graft. The cross-sectional center of both, the inner and outer layer of BC as well as the mesh was computed at different positions, and their deviation was calculated. For the X-ray tomography high-resolution analysis and three-dimensional rendering, phoenix nanotom^®^ m from GE Sensing and Inspection Technologies (Hürth, Germany), respectively, VGStudio MAX 2.1^®^ from Volume Graphics GmbH (Heidelberg, Germany) were used.

#### Scanning electron microscopy

Scanning electron microscopy (SEM) was applied to investigate the morphology of the inner and outer surfaces as well as the cross-sectional surface of the grafts. Samples were ultra-rapidly frozen in nitrogen slush and freeze-dried for 12.5 h. The dry samples were sputtered with 20 nm gold by an EM ACE 600 Sputter Coater^®^ from Leica (Austria) and analyzed by the Nova NanoSem 230^®^ from FEI (Netherlands).

### Mechanical characterization of the vascular graft

A different batch from the vascular grafts implanted was used to assess the mechanical properties of the generated BC vessels.

For longitudinal tensile testing, tubular samples of 4 cm in length were set to a tensile testing machine from MTS Systems Corporation (Eden Prairie, MN, USA). Each sample was stretched longitudinally with a constant rate of 15 mm/s, and displacement and force at the breaking point were recorded. Due to the BC reinforcement with an embedded support device, the stress at break was assigned to the mechanically superior mesh. To determine the force at the break of mechanically inferior BC layers, a force decrease of more than 0.1 N within 0.1 s was defined as significant for all tensile testing.

For circular tensile testing, tubular samples of 0.5 cm in length were stretched circularly with a constant rate of 15 mm/s.

For suture retention testing, tubular samples of 2 cm in length were sutured by 14 stitches with a stitch-edge distance of approximately 1 mm, with double-armed 7-0 non-resorbable Prolene^®^ from Ethicon (Bridgewater, MA, USA). The prepared samples were stretched longitudinally with a constant rate of 15 mm/s.

### Surgical procedure

#### Approval and standards

The research protocol was coordinated and approved by the national ethics committee of Israel under the national registration number IL-16-11-369. All facilities and activities at Lahav C.R.O. (D.N. Negev, Israel) were accredited and monitored in accordance with GLP and ISO 9001 (2015) standards for quality and service. This study was conducted in accordance with the Israeli National Council of Animal Experimentation. This study adhered to the ARRIVE Guidelines and was designed and performed under consideration of the 3Rs (Replace, Reduce, and Refine) principles for animal experimentation.

#### Animals

Eight healthy female pigs with a weight of 78–91 kg were purchased by Lahav C.R.O. (D.N. Negev, Israel). Standard diet (soft food) expanded for pigs from AMBAR Feed mill (Granot M.P. Heffer 3881100, Israel) were provided. Housing started at least 10 days prior to intervention to adapt the animals to the experimental environment.

#### Surgery

The selected pigs were fasted for 24 h before anesthesia to prevent vomiting. For preparation, all animals were washed, shaved, and disinfected. Preoperative antibiotics were administered in the usual manner. All surgical procedures were performed under aseptic conditions in an operating suite dedicated to veterinary surgery and under general anesthesia: Prior to the surgeries, 2 mg/kg xylazine (Anased^®^, AKORN, USA) + 10 mg/kg ketamine (Clorkeam^®^, Vetoquinol, France) were administered by intramuscular injection. Anesthesia was induced by inhalation of 3% isoflurane (Piramal Critical Care, USA) *via* a mask and intravenous administration of 5–10 mg diazepam per animal (Diazepam-Ratiopharm^®^, Ratiopharm, Germany). During surgeries, anesthesia was maintained by the administration of 1–3% isoflurane. Animals were intubated and ventilated *via* a ventilator, breathing parameters were constantly monitored (ECG, pulse oximetry, rectal temperature, blood pressure, and CO_2_). Antibiotic prophylaxis was administered using 0.1 ml/kg Pen&strep^®^ (200 mg/ml penicillin + 250 mg/ml streptomycin, Norbrook, USA). A single shot of 200 mg amiodarone (Cordarone, Pfizer Inc., USA) was administered i.v. to all animals preoperatively. After intubation, the animals were placed in a supine position with their legs attached to the operating room table. After conventional sternotomy, pericardiotomy was performed. The left anterior descending (LAD) artery was identified and ligated after 30 min of preconditioning (3 times for 5 min closure and 3 times for 5 min of flow using an elastic band and a tourniquet system). Within the first days after surgery, the animals were monitored routinely and further analgesia was given as necessary.

#### Off-pump coronary artery bypass grafting model

Coronary artery bypass grafting (CABG) was performed on the beating heart utilizing a stabilizer and an intracoronary shunt by two experienced cardiac surgeons. For routine anticoagulation during CABG, heparin was administered, aiming for an activated clotting time greater than 400 s, and later antagonized with protamine. The graft was implanted in the middle segment of the LAD distally to the occluded section. After completion of the anastomosis with the ascending aorta, intraoperative flow measurement (Medistim, Norway) was conducted to validate the patency of the graft. For antithrombotic prophylaxis, aspirin (100 mg/day) was administered daily during the postoperative period. Termination was performed 4 weeks post-implantation. Cardiac arrest was induced by injecting a 20% solution of pentobarbital (Pental Veterinary, CTS Chemical Industries, Israel).

#### *In vivo* assessments

The primary endpoint was graft patency after CABG and 4 weeks of follow-up determined by angiography and intraoperative flow measurement prior to model termination. The secondary endpoint was vascular remodeling defined by the presence of endothelial cells in the inner lumen.

### Primary endpoint

#### Postoperative assessment and follow-up

Right after the CABG procedure, coronary angiography was performed. Four weeks after implantation, an angiographic assessment was repeated, and thereafter, the grafts were explanted *in toto* with attached cardiac and aortic segments. Angiography was used to assess the patency of the grafts, in particular, grafts were classified as fully open or partially narrowed, or fully closed according to the established scoring system by Fitz Gibbon et al. for grading distal, proximal anastomoses in the early stage after CABG (13).

### Secondary endpoint

#### Histologic analysis

Samples from two grafts from both anastomotic sites and the middle section of the graft were prepared and embedded in paraffin, cross-sectioned, and stained with hematoxylin and eosin (HE staining) for histologic assessment. For immunofluorescence staining, the following antibodies were used: rabbit anti-laminin (ab11575, dilution at 1:200) and goat anti-smooth muscle actin (SMA) (ab7817, dilution at 1:400) from Abcam (UK), goat anti-vascular endothelial (VE) cadherin (sc6458, dilution at 1:100) from Santa Cruz Biotechnology (Dallas, US), mouse anti-CD31 (MCA1746GA, dilution at 1:100) from AbD Serotec (Germany), mouse anti-CD68 (BA4D5, dilution at 1:50) from AbD Serotec (Germany). All secondary antibodies were diluted 1:200 (Life Technologies, USA). Cell nuclei were stained with 4’,6-diamidino-2-phenylindole (DAPI) (0.125 mg/ml) (Sigma-Aldrich, Germany).

## Results

### *In vitro* characterization of the vascular graft

**FIGURE 1:**
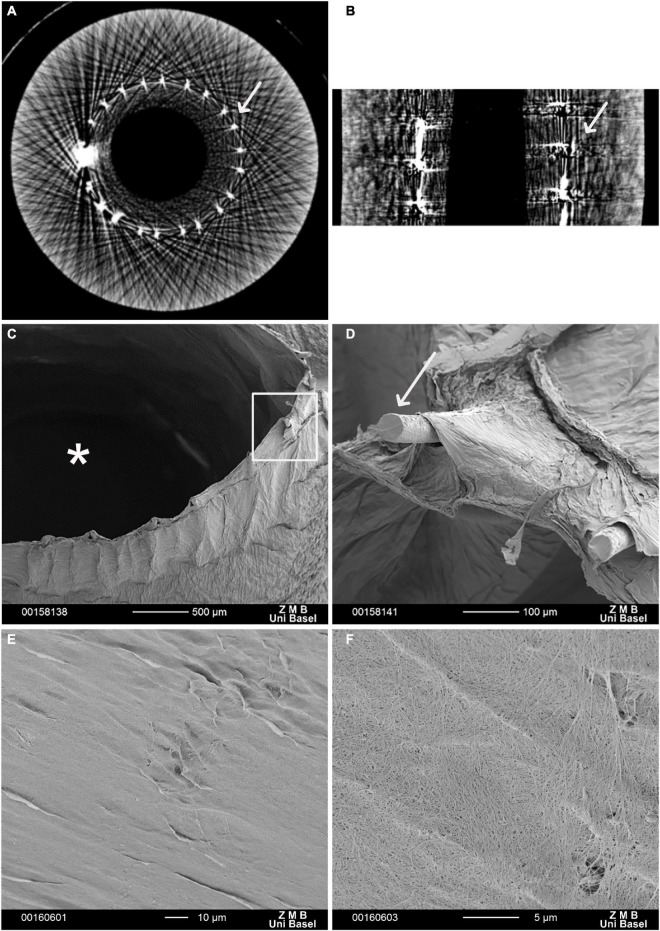
Electron microscopy of a vascular graft. Micro-computed tomography (Micro-CT) scan. **(A)** Cross section, **(B)** longitudinal section of the reinforced bacterial cellulose (BC)-based vascular graft. White arrows indicate the Co-Cr mesh scanning electron microscopy (SEM). **(C)** In the cross-section, the structure of the graft comprising an inner BC layer defining the graft lumen (*), the embedded metallic mesh, and an outer BC layer is visible. **(D)** The enlargement (rectangular frame) shows the ends of the mesh wires (arrow). These protruding ends result from cutting and further processing of the vascular graft. **(E,F)** BC-vascular graft luminal surface. **(F)** At higher magnification, porosity of BC-vascular graft luminal surface.

All grafts met the predefined criteria, particularly, having a length of 20 cm and an inner diameter of 3 mm, a wall thickness of 2.73 ± 0.21 mm, and an outer diameter of 9.45 ± 0.43 mm (Table 1). Micro-CT revealed a homogenous inner layer of BC and centralization of the mesh along with the graft (Figures 1A,B). BC was combined with a highly flexible and kink-resistant cobalt–chromium mesh embedded within the graft wall. The mesh was covered entirely from both sides by BC without an external connection (Figures 1C,D). Furthermore, SEM revealed an even luminal porous surface (Figures 1E,F).

**TABLE 1:** Bacterial cellulose (BC)-vascular graft morphological and mechanical features (*n* = 3).

Morphological properties				
BC graft inner diameter	BC graft outer diameter	CoCr mesh diameter	BC graft length	BC graft wall thickness
3 mm	9.45 ± 0.43 mm	5 mm	20 cm	2.73 ± 0.21 mm
**Mechanical properties**				
Longitudinal tensile test		Circumferential tensile test		Suture retention test
2.85 ± 2.08 N		0.98 ± 0.42 N		8.83 ± 1.47 N

Longitudinal and circumferential tensile testing resulted in a force at break of 2.85 ± 2.08 N and 0.98 ± 0.42 N, respectively. The suture retention testing resulted in a force at break of 8.83 ± 1.47 N.

### *In vivo* coronary artery bypass grafting pig model

Intraoperatively, the surgical handling of the graft was reported comparable to arterial grafts in terms of elasticity and wall rigidity. The vessel was blood tight when anastomosed and did not show any effect of kinking or twisting. The artificial “adventitia” stayed adherent and did not show any signs of delamination. The BC-vascular graft handling has been evaluated by two experienced cardiac surgeons.

**FIGURE 2:**
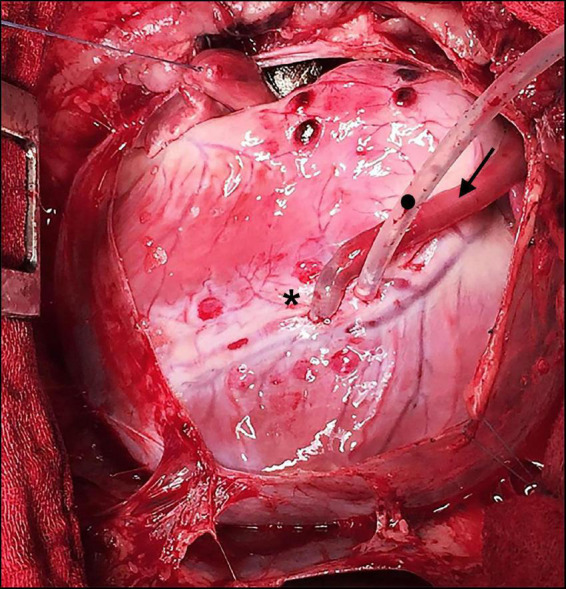
Intraoperative situs during off-pump coronary artery bypass grafting (CABG). Bacterial cellulose (BC) graft as a coronary bypass (arrow in direction of flow) after distal anastomosis to the occluded left anterior descending (LAD) (*) using an elastic band with a tourniquet (•).

There were no complications arising from neither the BC nor the embedded CoCr mesh and no bleeding from the anastomotic site has been observed after the reversal of heparin. The graft presented visible pulsation without signs of rupture or dissection once it was de-clamped (Figure 2 and Supplementary Video 1).

### Postoperative assessment and follow-up

**FIGURE 3:**
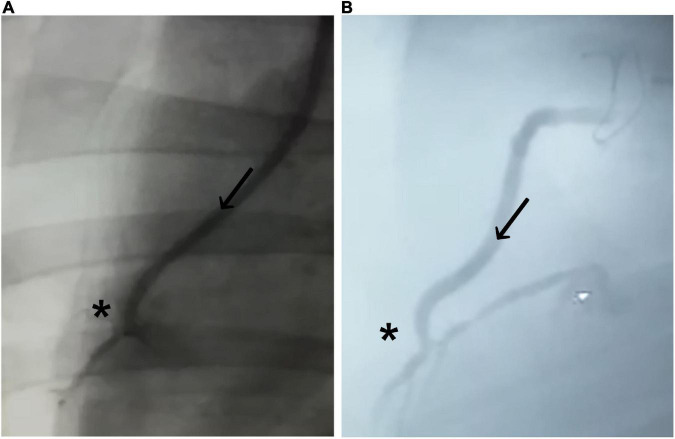
Coronary angiogram of the implanted graft. **(A)** Immediately after implantation and **(B)** 4 weeks after implantation. Bacterial cellulose (BC)-vascular graft shows good postoperative results with a complete patency profile as coronary artery bypass and antegrade flow through the BC graft (arrow in direction of the flow) toward the distal anastomosis (*).

The initial postoperative angiography conducted directly after finishing the CABG procedure revealed sufficient graft patency of the implanted BC graft in all eight animals. There were no signs of graft dissection or narrowing at the distal anastomotic site (Figure 3A). All animals recovered within 24 h, consuming solid food and water 24 h after surgery.

Coronary angiography 4 weeks after CABG confirmed sufficient graft patency without signs of graft dissection or burst in all animals (Figure 3B; Table 2). None of the animals showed a completely closed lumen of the graft, dissection, ruptures, or bleeding during the 4-week period when evaluated with angiography.

**TABLE 2:** Coronary angiography 4 weeks after coronary artery bypass grafting (CABG) (scoring).

Patency-scoring	Graft 1	Graft 2	Graft 3	Graft 4	Graft 5	Graft 6	Graft 7	Graft 8
100% opened								
> 75% opened								
< 75% opened								
100% closed								

Grey boxes are indicating the patency scoring level of each graft.

### *In vivo* cellularization of the vascular grafts

**FIGURE 4:**
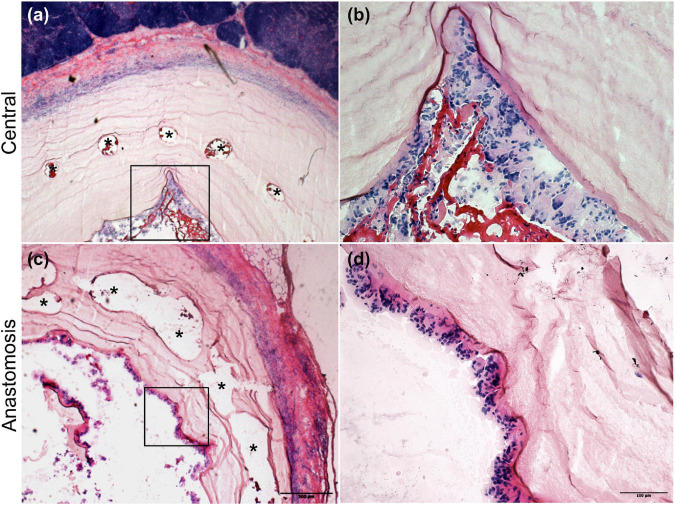
H&E staining of the explanted grafts 4 weeks after implantation. The explanted Bacterial cellulose (BC)-vascular graft reveals a three-layered wall structure indicating remodeling by host cells. Representative images of the vascular grafts at the central part **(a,b)** and at the anastomosis site **(c,d)** at high 20 × **(b,d)** and low 4 × **(a,c)** magnifications. *indicates the empty holes left from the embedded mesh, which were removed during the histological process.

**FIGURE 5:**
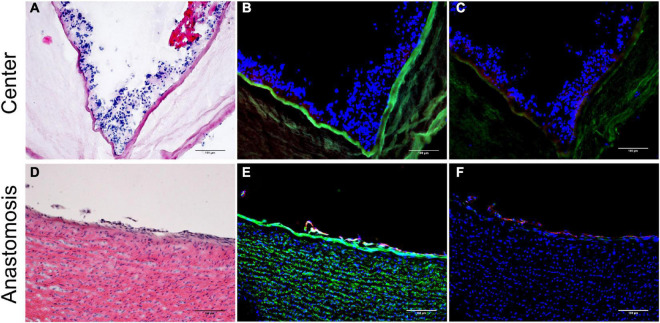
Inner layer of the vascular grafts 4 weeks after implantation. Representative images of the vascular grafts at the central part **(A–C)** and at the anastomosis site **(D–F)** stained with H&E **(A,D)** and immunofluorescence antibodies specific for Ve-Cadherin (red), α-SMA (cyan), laminin (green) **(B,E)**, and for CD31 (red) and CD68 (green) **(C,F)**. Nuclei were stained with DAPI (blue) **(B,C,E,F)**. Scale bar = 100 μm.

Four weeks after implantation, the cobalt–chromium mesh appeared still centered and embedded into the BC layers. BC layers were still visible (Figure 4). However, it was possible to observe the presence of SMCs and ECs covering the BC-vascular graft luminal surface suggesting a possible remodeling of the graft in the long term (Figure 5). The remodeling of the graft and the endothelialization were more evident in the anastomotic sites compared to the center of the graft following 1 month upon implantation (Figures 4, 5). Macrophages have been not detected (Figures 5C,F). Only in one case of endocarditis, inflammatory cells were found within the implant.

**FIGURE 6:**
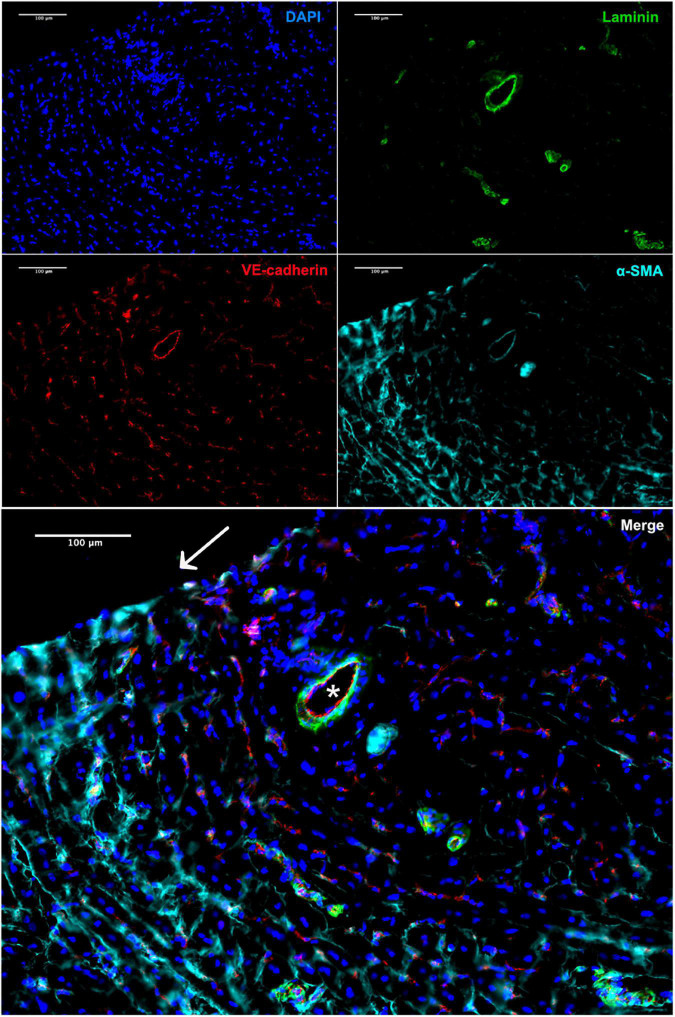
Immunofluorescence staining of the explant 4 weeks after implantation. Vasa privata (*) within the remodeled BC-vascular graft wall (anti-VE-cadherin in red, anti-αSMA in cyan, and anti-laminin in green). Nuclei were stained with DAPI. Scale bar = 100 μm.

The presence of *vasa privata*, namely blood vessels belonging to a specific organ, nourishing, and perfusing it, was investigated. Functional *vasa privata* through the vascular wall were seen in the analyzed grafts (Figure 6). The *vasa privata* were composed of clear endothelial and smooth muscle cell layers and vascular basal membrane as shown by the presence of laminin.

Besides, there were no signs of thrombosis or luminal narrowing as seen in the case of neointimal hyperplasia.

## Discussion

Due to rising life expectancy and the increasing prevalence of vascular diseases, there is a growing need but limited availability of autologous vascular grafts for surgical revascularization. In the context of peripheral and CABG, TEVGs made of biomaterials appear to be promising alternatives. However, complex manufacturing of particularly small-diameter grafts of sufficient length and their limited long-term *in vivo* patency appears to be challenging and represents the limiting factors for their clinical introduction. Consequently, the aim of this study was to generate a novel BC-vascular graft of sufficient length for CABG procedures, precisely greater than 15 cm featuring a small inner diameter of less than 6 mm. Through various improvements, the herein described grafts overcome various limitations of previously reported biological grafts: short and simple production, sufficient mechanical strength, and a satisfactory length of 20 cm for cardiovascular applications. The main aim of the graft design was to allow the *in vivo* endothelialization process to avoid any time-consuming pre-seeding or cell expansion step. Beyond manufacturing and *in vitro* characterization, this was the first attempt to implant a BC-vascular graft as a coronary artery bypass graft in a large animal ischemic heart model.

In the past, one of the first BC small-diameter vascular grafts was described in 2001. Although much smaller with a luminal diameter of only 1 mm and inferior to the herein described grafts regarding mechanical strength, the great potential of BC grafts for cardiovascular applications was demonstrated. The microvascular graft was implanted as carotid interponat in a small-animal model and remained patent for 4 weeks (14). Since then, the methods of graft production and biomechanical characteristics have been continuously improved.

Among various features of BC-vascular grafts, burst pressure is a key indicator of mechanical strength and clinical applicability. Bodin et al. have shown that BC-vascular graft burst pressure strength increased at an oxygen ratio of 100% compared to a ratio of 21, 35, or 50%. This is most likely because BC-producing bacteria are obligate aerobes and produce more BC with higher density under more favorable environmental conditions (15). Comparing the burst pressure of different vascular grafts, native BC grafts compete with autologous venous grafts but not with arterial grafts. The latter is characterized by a thicker wall, physiologically withstanding higher blood pressure in the arterial system (16, 17). For saphenous vein grafts, a burst pressure of about 1,600 mmHg has been reported. Internal mammary artery grafts withstand a pressure higher than 3,100 mmHg (16). After CABG procedures, venous grafts, physiologically designed to withstand low pressure within the venous system, have to withstand high pressure within the arterial system. Insufficient wall strength of venous grafts favors micro lesions leading secondarily to intimal hyperplasia. Reactive migration and proliferation of vascular SMCs result in an extracellular matrix of connective tissue, consecutively, leading to graft stenosis and occlusion (18). In general, intimal hyperplasia is considered a major cause of graft failure (19). Therefore, BC grafts have to fulfill specific requirements for compliance and mechanical strength. Since all the herein described grafts remained patent for over 4 weeks, sufficient compliance and mechanical strength are presumed.

As highlighted above, coronary angiography 4 weeks after CABG revealed complete patency. To the best of our knowledge, this was the first time that BC grafts were implanted as coronary bypass grafts in a large animal model. So far, BC grafts were tested prevalently as interponat in the common carotid artery in various animal models. This is due to the favorable cervical accessibility and the possibility of implanting short grafts of a few centimeters and even millimeters. Wippermann et al. performed carotid replacement in a large animal model. For carotid interposition, grafts up to 10 mm in length with an inner diameter of 3.0–3.7 mm were applied in eight domestic pigs. After 3 months, a patency of 87.5% was reported. While one graft was found occluded due to collapsed vascular walls, the others showed no signs of thrombosis, dilatation, dehiscence, or aneurysm formation (10). Malm et al. tested BC grafts with an increased length in a long-term animal study. Samples up to 40 mm length and an inner diameter of 4 mm were implanted bilaterally in the common carotid arteries of eight sheep. Five cases of acute thrombosis and severe anemia occurred in the early postoperative period. From the three-remaining sheep, all grafts remained patent after 3 months and five of six, respectively, 83.3% after 6 months. The sheep with unilateral occlusion had to be euthanized after 8 months revealing an organized thrombosis and signs of intimal hyperplasia in the perianastomotic region. Thirteen months after implantation the grafts from the remaining two sheep were explanted revealing 75% patency (11). Although various experimental studies display the great potential of BC grafts for a broad field of cardiovascular application, there has been no clinical translation yet. This requires even better long-term results without thrombotic complications.

As mentioned before, the grafts were explanted after 4 weeks. According to our findings, the explanted grafts were well-integrated into the surrounding host tissue without signs of inflammation. All of them revealed a native-like three-layered wall structure showing a layer of ECs with the basal lamina, a concentric layer of SMCs, and an outer layer of fibroblasts. The presence of various cell types and the occurrence of *vasa privata* confirm the suitability of the BC fibril network as a scaffold for cellularization (20, 21). Since the histological findings indicate sufficient graft remodeling within 4 weeks, the pre-procedural step of cell harvesting, expansion, and seeding prior to implantation is unnecessary in this graft type. This represents an important feature in terms of clinical translation as these three steps are time-consuming, and time the patients do not have prior to a CABG procedure. Nevertheless, it seems very important to gain a better understanding of *in vivo* cellularization dynamics and conditions in order to create an optimal graft environment. By continuous optimization of BC grafts such as surface modification, *in vivo* endothelialization might be further sped up.

The study has one major limitation. Since the main aim was to implant the BC-vascular graft as a coronary bypass graft in a large animal model as proof of concept, *in vivo* testing was carried out only in eight domestic pigs without a control group as a proof-of-concept study. Larger and controlled animal studies are required to verify the results, especially in the medium-and long-term.

In conclusion, small-diameter BC-vascular grafts with an application-oriented length of more than 15 cm can be generated. Furthermore, these grafts reveal great potential when applied as a coronary bypass graft in an ischemic heart model in pigs. The grafts showed a three-layered structure composed of an inner endothelial layer with SMCs beneath and *vasa privata* formation. In the future, more animal studies are needed to determine the potential of BC-vascular grafts for cardiovascular applications beyond CABG. In case of continued successful *in vivo* testing, coronary and peripheral artery bypass grafting with BC grafts should be evaluated.

## Data availability statement

The original contributions presented in this study are included in the article/Supplementary material, further inquiries can be directed to the corresponding author.

## Ethics statement

The research protocol was coordinated and approved by the National Ethics Committee of Israel under the national registration number IL-16-11-369. All facilities and activities at Lahav C.R.O. (D.N. Negev, Israel) were accredited and monitored in accordance with GLP and ISO 9001 (2015) standards for quality and service. This study was conducted in accordance with the Israeli National Council of Animal Experimentation. This study adhered to the ARRIVE Guidelines.

## Author contributions

BW: concept or design of the work. MG and BW: acquisition of data. FM, DF, AM, BP, and BW: analysis and interpretation of data and drafting the manuscript. All authors revising the manuscript critically for important intellectual content and approved of the version of the manuscript to be published.

## Supplementary material

Supplementary Figure 1 Customized bioreactor used for bacterial cellulose (BC)-vascular graft production. **(A)** Bioreactor scheme consists of a culture chamber filled with bacteria suspension, four rotating steel shafts covered by a silicon tube, and four stepper motors. **(B)** Picture of the bioreactor during the fermentation process.
