# Supplementary figures and images for: Small-diameter bacterial cellulose-based vascular grafts for coronary artery bypass grafting in a pig model
Source: Front Cardiovasc Med. 2022 Sep 26;9:881557. doi: 10.3389/fcvm.2022.881557 (PMC9548626; doi:10.3389/fcvm.2022.881557)

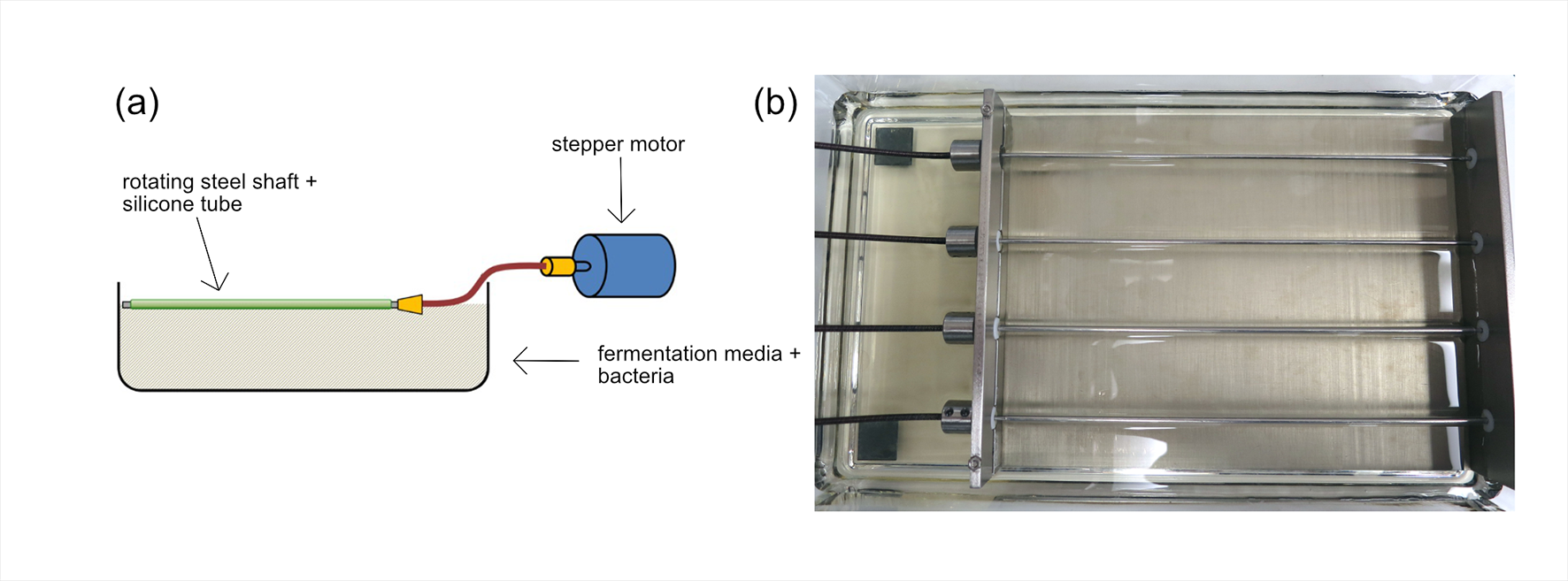

Supplement: Supplementary Figure 1 — Customized bioreactor used for bacterial cellulose (BC)-vascular graft production. (A) Bioreactor scheme consists of a culture chamber filled with bacteria suspension, four rotating steel shafts covered by a silicon tube, and four stepper motors. (B) Picture of the bioreactor during the fermentation process. [file Image_1.TIFF]
